# Tuberculosis and the Research Workforce Renewal Crisis in Brazil: How Can We Prevent a Future Without New Researchers?

**DOI:** 10.1590/0037-8682-0133-2025

**Published:** 2025-11-03

**Authors:** Beatriz Barreto-Duarte, Klauss Villalva-Serra, Ricardo Alexandre Arcêncio, Julio Croda, Ethel Leonor Noia Maciel, Afrânio Lineu Kritski, Bruno Bezerril Andrade

**Affiliations:** 1 Laboratório de Pesquisa Clínica e Translacional, Fundação Oswaldo Cruz, Salvador, BA, Brasil.; 2 Instituto de Pesquisa em Populações Prioritárias (IRPP), Instituto MONSTER de Ensino, Assistência, Pesquisa e Desenvolvimento Tecnológico em Saúde, Salvador, BA, Brasil.; 3 Instituto Monster de Ensino, Assistência, Pesquisa e Desenvolvimento Tecnológico em Saúde, Salvador, BA, Brasil.; 4 Rede Brasileira de Pesquisas em Tuberculose (REDE-TB), Rio de Janeiro, RJ, Brasil .; 5 Universidade de São Paulo, Escola de Enfermagem, Ribeirão Preto, SP, Brasil.; 6 Fundação Oswaldo Cruz, Campo Grande, MS, Brasil.; 7 Universidade Federal do Mato Grosso do Sul, Faculdade de Medicina, Campo Grande, MS, Brasil.; 8 Universidade Federal do Espírito Santo, Departamento de Enfermagem, Vitória, ES, Brasil.; 9 Universidade Federal do Rio de Janeiro, Faculdade de Medicina, Rio de Janeiro, RJ, Brasil.

**Keywords:** Tuberculosis, Brazil, Scientific Research

## Abstract

Tuberculosis (TB) is among the oldest and deadliest infectious diseases, particularly when associated with human immunodeficiency virus (HIV) and antimicrobial resistance. Despite the progress in prevention, diagnosis, and treatment, global elimination remains elusive and is driven largely by socioeconomic inequalities and systemic challenges. Although scientific research is a cornerstone of the WHO End TB Strategy, it has been chronically underfunded and undervalued in Brazil’s health agenda. One critical consequence is the weakening of pipelines for future TB research. Funding shortages, lack of incentives, and the shifting attention toward s other emerging diseases have made it increasingly difficult to recruit and retain scientists in the field of TB. This opinion paper aimed to explore the historical role of Brazilian science in advancing TB control while addressing the emerging crisis of renewing the country’s TB scientific workforce. We conducted a narrative synthesis of the available literature, reviewing impactful peer-reviewed articles produced by Brazilian scientists in the field of TB science, alongside official documents from the Brazilian Ministry of Health. This was complemented by a bibliometric analysis of the output of TB-related PhD theses (2016-2024) and PubMed-indexed publications (2001-2024) from Brazilian institutions. Finally, we discuss the systemic barriers affecting early career researchers and outline strategies for revitalizing interest and sustaining scientific progress. These include targeted TB research funds through public-private partnerships, structured mentorship programs, and competitive early career fellowships. Such interventions are essential for reversing the current decline in TB research engagement and ensuring that Brazil continues to contribute to global TB elimination efforts while preserving its scientific legacy.

## HISTORY OF TB SCIENCE IN BRAZIL

**FIGURE 1:**
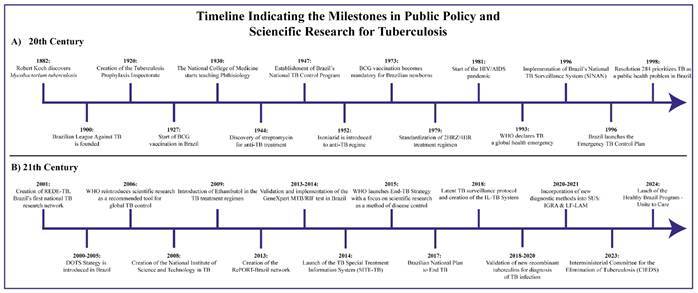
Timeline indicating the milestones in public policy and scientific research on tuberculosis.

**Legend:** Key historical milestones in tuberculosis research and control. **1882:** Robert Koch discovers *Mycobacterium tuberculosis*, revolutionizing our understanding of the disease. **1900:** Foundation of the Brazilian League against Tuberculosis, the first major national initiative to combat TB. **1921:** Development of the Bacillus Calmette-Guérin (BCG) vaccine in France. **1944:** Discovery of streptomycin, the first effective antibiotic against TB. **1947:** Establishment of Brazil’s National Tuberculosis Control Program (PNCT). **1973:** BCG vaccination becomes mandatory for newborns in Brazil. **1980s:** The HIV/AIDS epidemic leads to a sharp increase in the number of global TB cases. **1993:** WHO declares TB a global emergency and recommends Directly Observed Treatment (DOTS). **1996:** Brazil launches the Emergency TB Control Plan. **1998:** Resolution No. 284 prioritizes TB as a public health issue in Brazil. **2000:** Amsterdam Declaration, a landmark agreement among ministers from 20 high-burden TB countries establishing a global action framework for TB strategies. **2001:** Creation of REDE-TB, Brazil’s first national TB research network. **2003:** Mandatory HIV testing is recommended for patients with TB in Brazil. **2003**: Launch of Stop TB Brazil, a national partnership to end TB^101^. **2012:** Start of a Parliamentary Front for the Fight Against Tuberculosis^102^. **2013**: Brazil joins RePORT International, a global TB research network. **2014**: Implementation of Xpert MTB/RIF rapid molecular test in Brazil’s public health system (SUS). **2017**: Brazil participates in establishing the BRICS TB Research Network. **2018**:1^st^ UN High-Level Meeting on TB^103^. **2019**: Launch of joint Operational Instruction No. 1, integrating Brazil’s social assistance network with the healthcare system for TB control. **2023**: The WHO Director-General established a TB Vaccine Accelerator Council in 2023 to facilitate the development, testing, authorization, and use of new TB vaccines, drawing on lessons learned from the response to the COVID-19 pandemic^104^. **2023**: Creation of Brazil’s Interministerial Committee for the Elimination of Tuberculosis and Other Socially Determined Diseases (CIEDS). **2023**: Portaria GM/MS nº 2.259, de 8 de dezembro de 2023 - Institui o Programa de Produção e Desenvolvimento Tecnológico para Populações e Doenças Negligenciadas - PPDN. **2023**:2^nd^ UN High Level Meeting on TB^105^. **2023**: Portaria GMMS nº 3.089, de 15 de janeiro de 2024 - Altera a Portaria GM/MS nº 2.262, de 8 de dezembro de 2023, que institui o Programa para Ampliação e Modernização de Infraestrutura do Complexo Econômico-Industrial da Saúde - PDCEIS^106^. **2024**: High-Level Meeting on antimicrobial resistance 2024 (TB included as a priority)^107^. **2024**: Establishment of the Brasil Saudável - Unir para Cuidar Program targeting a TB incidence of < 10 cases per 100,000 inhabitants. **2024**: The 7th Global Forum on TB Vaccines, held in Rio de Janeiro (October 2024), reaffirms the global commitment to develop a more effective TB vaccine than BCG by 2027^51^.

Despite advances in scientific research and the development of new control policies, tuberculosis (TB) remains a major public health challenge worldwide^1^. From Robert Koch’s discovery of *Mycobacterium tuberculosis* in 1882 to modern innovations, the continuing fight against TB underscores the critical role of scientific and governmental initiatives in lightening the disease burden^1^ ^,^ ^2^. Throughout the 20th and 21st centuries, Brazil played a pivotal role in developing TB control strategies, including the establishment of the Brazilian League Against Tuberculosis in 1900, implementation of the National Tuberculosis Control Program (PNCT) in 1947, and mandatory administration of the Bacillus Calmette-Guérin (BCG) vaccine to newborns in 1973^3^ (Figure 1).

Despite achievements in controlling other infectious diseases during the 20th century, such as the development of vaccines that enabled the eradication of urban yellow fever and the elimination of smallpox in 1980, their transformative impact has not been directly mirrored in the fight against TB. Thus, the persistent burden of TB as a severe public health issue, coupled with the disproportionate attention given to it compared to other emerging diseases, has been considered a tolerated failure, leading to the classification of TB as a neglected disease^4^.

Nevertheless, during the 1980s and the 1990s, the HIV/AIDS epidemic significantly increased the global TB incidence, prompting the World Health Organization (WHO) to declare a global TB emergency in 1993 and recommend the adoption of the Directly Observed Treatment, Short-course (DOTS)^5^. In response, Brazil launched the Emergency Plan for TB Control in 1996, closely followed by Resolution No. 284, which prioritized TB as a public health issue, in 1998^3^ ^,^ ^6^. This period also marked the introduction of mandatory HIV testing for patients with TB, reinforcing the need for an integrated disease control approach. Nevertheless, there was still no national TB research agenda, and public health decisions were often not based on scientific evidence (Figure 1).

In 2001, with funding from the National Council for Scientific and Technological Development (CNPq), the Brazilian Tuberculosis Research Network (REDE-TB) was established, which helped identify WHO priorities in operational research and enabled the definition of a new national research agenda while also boosting national scientific innovation^7^ ^-^ ^9^. This initiative fostered greater interaction between scientific researchers and the government, leading to the formation of the National Technical Advisory Center under the National TB Program^3^ ^,^ ^6^. Additionally, Brazil’s active involvement in global research networks such as the RePORT-International Consortium (2013)^10^ and the BRICS TB Research Network (2017)^11^ has promoted multinational scientific collaboration aimed at improving TB elimination^12^ (Figure 1).

More recently, Brazil has experienced a post-COVID rebound in TB burden, with increasing rates of both incidence and mortality since 2022. Consequently, new policies have been drafted in response to these issues and the Interministerial Committee for the Elimination of Tuberculosis and Other Socially Determined Diseases (CIEDS) was established in 2023, thus reinforcing Brazil’s commitment to eliminating the disease with a special focus on the most vulnerable populations. In 2024, the *Brasil Saudável - Unir para Cuidar* (Healthy Brazil - Unite to Care) Program was introduced, bringing together 14 ministries alongside civil society^13^ to drive the TB incidence towards fewer than 10 cases per 100,000 inhabitants and addressing the social inequalities that sustain transmission. This initiative has already allocated $20 million for expanded testing, active case finding, and preventive-therapy scale-up^14^, further supporting the increases in Xpert® MTB/RIF coverage and uptake of short-course, rifapentine-based preventive regimens from 2022-2024^15^ (Figure 1).

Notably, Brazil has established itself as a leader in TB research in Latin America^16^ with institutions affiliated to REDE TB, such as the Federal University of Rio de Janeiro and the Oswaldo Cruz Foundation, which play central roles in developing new technologies and approaches for evaluating public health interventions^17^ ^,^ ^18^. The impact of Brazilian scientific discoveries has been recognized globally, with the WHO highlighting REDE-TB as a model for integrating national TB programs with scientific research networks and civil society^3^ ^,^ ^6^.

In this study, we aimed to highlight Brazil’s TB research output by examining peer-reviewed publications and national policy documents so as to map how scientific achievements were pivotal in improving Brazil’s nationwide TB control strategies. We then discuss how persistent barriers have resulted in decreasing interest in TB science among early career researchers, thereby threatening Brazil’s journey towards a TB-free future. Finally, by proposing targeted strategies, including dedicated TB research funds, expanded mentorship networks, and public-private partnerships, we aimed to assist in identifying pathways for reinvigorating the younger generation of Brazil’s TB research workforce and sustain science-driven gains in TB management.

## METHODS

### Study design and scope

This paper presents a scientific opinion piece focused on the dilemma of how, despite the impact of Brazil’s involvement in TB research on the country’s public health efforts towards disease control, the current decline in interest among young Brazilian scientists in TB science presents a substantial threat towards a TB-free future. To combat this, we present possible strategies towards increasing interest among the younger generation in creating a TB-targeted workforce and driving scientific advancements that can further improve the fight against TB in Brazil. 

This study aimed to synthesize the key Brazilian scientific advancements that have affected TB public health control. To this end, we reviewed a plethora of impactful Brazilian articles published in major biomedical literature databases, including PubMed, EMBASE, and SciELO, without restrictions on the publication period or language. Additionally, we examined policy documents from the epidemiological reports of the Brazilian Ministry of Health and action plan guidelines obtained directly from the Ministry’s public repository. The insights derived from this review were further enriched by the perspectives of renowned and experienced Brazilian TB researchers. 

Furthermore, bibliometric trend analysis was also performed using data from the Brazilian PhD Thesis Registry database (2016-2024)^19^, using Mann-Kendall and Theil-Sen tests to detect temporal trends in TB-focused theses per year. Similar analyses were conducted to assess the research output of authors associated with Brazilian institutions. To do so, we extracted the total number of PubMed-indexed articles on TB, with at least one author associated with Brazilian institutions, ranging from 2001 to 2024. The search query was defined by the search terms “Tuberculosis” OR “Mycobacterium tuberculosis” OR (“TB” OR “Tuberculose” AND (Brasil[Affiliation] OR Brazil[Affiliation])), with no language restrictions.

## RESULTS

### Scientific Achievements with Impact on Public Health

Among Brazil’s scientific notable contributions, the following stand out:

### Advancements in TB Diagnosis

For over a century, chest radiography (CXRs) has been the primary method for diagnosing TB in Brazil. However, its limitations, including low diagnostic specificity, logistical difficulties in remote areas, and the initial dependence on physicians for diagnosis, have driven the search for new diagnostic technologies^20^ ^-^ ^22^. Consequently, numerous studies have focused on developing, validating, and implementing innovative methods within the Brazilian Unified Healthcare System (SUS).

A landmark example is the adoption of GeneXpert MTB/RIF in Brazil. Before its official implementation in the SUS, multicenter studies compared its performance with that of smear microscopy, and conducted economic analyses to assess the costs associated with this new technology^23^ ^,^ ^24^. Additionally, a pilot study conducted in two high-incidence cities demonstrated the benefits of replacing smear microscopy, thereby laying the foundation for the creation of the Rapid TB Testing Network^25^. Subsequent research confirmed that this evidence-based adoption strategy was successful, as it had a positive impact on accelerating TB diagnosis, leading to a 9.7% increase in the total reported TB cases, with a particular focus on drug resistance screening resulting in a 63.6% increase in the detection of confirmed drug-resistant TB cases at the national level^20^ ^,^ ^23^ ^,^ ^26^. Similarly, the clinical impact of using the Line Probe Assays, GeneXpert MTB/RIF, and GeneXpert ULTRA under field conditions was also evaluated^27^ ^-^ ^29^.

Implementation science has become a crucial tool for improving the TB healthcare system in Brazil. Studies identifying operational and logistical challenges in deploying Interferon-Gamma Release Assays (IGRAs) at the Tropical Medicine Foundation in Manaus^30^, along with those performing cost-benefit analyses, have contributed to the development of the Ministry of Health guidelines^30^ ^-^ ^32^. 

Other innovations include intensified research on biomarkers for TB infection and disease, further reinforcing Brazil’s commitment to innovation and diagnostic efficiency^10^ ^,^ ^33^ ^-^ ^35^. Furthermore, studies have focused on the development and validation of new biomarkers for the early diagnosis of latent and subclinical TB, as well as the accuracy of globally available rapid diagnostic tests^36^ ^-^ ^38^.

## PREVENTION

### Tuberculosis Preventive Therapy (TPT)

Trials conducted in Brazil have pioneered the demonstration of the efficacy of TB preventive therapy (TPT) in high-risk groups, including people living with HIV^39^, and provided foundational evidence for developing and validating novel TPT regimens worldwide^40^ ^,^ ^41^.

Historically, TPT in Brazil was based on a long regimen involving the daily administration of isoniazid for six to nine months, which was the primary preventive strategy until 2018^42^ ^,^ ^43^. However, this extended treatment often results in poor patient adherence. To optimize TPT, clinical trials conducted in Brazil have confirmed the efficacy of new regimens that reduce both treatment duration and number of required doses^44^. Seminal studies conducted in Brazil evaluated the efficacy of shorter and more patient-friendly treatment regimens in both people living with HIV and household contacts of patients with TB, demonstrating comparable efficacy to older regimens with markedly improved adherence^40^ ^,^ ^41^ ^,^ ^45^. 

These studies laid the groundwork for breakthrough implementation of the 3HP regimen in Brazil in 2021, which consists of isoniazid and rifapentine administered weekly for three months. This regimen demonstrated high efficacy in preventing the progression of TB infection to active disease, and was incorporated into national guidelines owing to its improved adherence rates and lower toxicity risk^40^ ^,^ ^41^ ^,^ ^44^. In parallel, the *ExpandTPT* project, which was launched in 2020 by the REDE-TB in collaboration with the Ministry of Health, has been crucial for expanding access to preventive therapy and training healthcare professionals to identify and treat patients eligible for TPT^46^.

Collaborations between the Ministry of Health and the scientific community, including the *ExpandTPT* project, have also assisted in Brazil’s swift rollout of the 3HP regimen. During implementation, TB researchers were requested to train and instruct providers about the advantages of the shorter course, significantly contributing to the substantial 189% increase in 3HP coverage from its implementation in 2021-2022, until the most recent 2024 reports, in which 3HP represented 76.4% of all TPT prescriptions^15^ ^,^ ^47^. 

## VACCINES

For over a century, BCG was the primary tool for TB immunization. However, its effectiveness in preventing pulmonary TB in adults remains limited. Brazilian studies have analyzed the efficacy of BCG revaccination in certain high-risk groups. Seminal trials include the BCG-REVAC cluster-randomized trial and the more recent study by dos Santos et al., which showed that BCG revaccination did not significantly reduce the risk of TB infection in school-aged children and in healthcare workers, highlighting the need for more effective prevention strategies^48^ ^,^ ^49^.

Brazil’s active involvement in international research networks has facilitated the development of more effective vaccines, including the M72/AS01E vaccine, which has demonstrated significant protection in Phase II clinical trials. Additionally, Brazilian research centers have participated in the evaluation of booster vaccines such as VPM1002 and MTBVAC, which aim to enhance immune responses and reduce disease transmission^50^. Notably, the 7th Global Forum on TB Vaccines held in Rio de Janeiro in 2024-the event’s first edition in the Americas-reinforced the urgency of rethinking vaccination strategies and investing in new approaches to overcome the limitations of BCG^51^. 

Finally, the introduction of genetic engineering technologies and mRNA-based vaccine platforms, such as those developed for COVID-19, has emerged as a promising strategy^52^. Advances in new therapies and vaccine research in Brazil further reflect the country’s commitment to TB elimination by combining local efforts with international collaboration to accelerate the introduction of new interventions into the healthcare system. Sustained investment in vaccine research and development is essential for reducing the disease burden and mitigating the impact of TB, particularly among vulnerable populations.

## TREATMENT

The standard regimen for drug-sensitive active TB in Brazil follows the WHO guidelines, consisting of an intensive phase with rifampicin, isoniazid, pyrazinamide, and ethambutol (RHZE), followed by a continuation phase with rifampicin and isoniazid alone^42^. National studies have explored shortened regimens to reduce the treatment duration and improve patient adherence.

Moreover, increasing resistance to TB medications has necessitated the adoption of new therapeutic approaches. Brazil has played a key role in the development of strategies for managing drug-resistant TB (DR-TB)^53^. National clinical trials have assessed the efficacy of bedaquiline and pretomanid, two innovative drugs incorporated into DR-TB treatment regimens, resulting in significantly shortened therapy duration and improved treatment success rates^54^ ^,^ ^55^. By adopting and studying new therapies, Brazil has strengthened its commitment to combating DR-TB, thus ensuring access to innovative medications and enhancing treatment outcomes.

Furthermore, recent experimental studies have demonstrated the efficacy of long-acting injectable bedaquiline administered in combination with 2-4-week oral companion regimens, as a promising option for ultrashort TPT regimens. Various bedaquiline-based regimens showed equivalent or even superior sterilizing activity compared with isoniazid and rifapentine administered daily for 28 days (1HP) in BCG-immunized BALB/c mice^56^. The development of these long-acting injectable formulations for TPT could substantially reduce the duration of therapy, thereby improving treatment completion rates worldwide.

### Directly Observed Therapy (DOT)

Directly Observed Therapy (DOT) is a public health strategy designed to ensure treatment adherence in patients with TB and reduce loss to follow-up. It involves the supervised administration of TB medications by healthcare professionals, who monitor patients’ treatment to ensure its proper completion^42^ ^,^ ^57^. It was implemented in Brazil in 1999. Subsequently, early studies conducted in Rio de Janeiro in the 2000s showcased the true effectiveness of DOT in Brazil, particularly in high-risk environments^58^ ^-^ ^60^. Other studies have highlighted the possibility of implementing DOT by engaging family members, volunteers, or even members of the community^61^.

Although the WHO has discontinued its recommendation of DOT as a global standard therapeutic approach, Brazil has continued to implement it, considering the strong evidence of its positive impact on treatment outcomes, particularly among vulnerable populations. Studies have shown that DOT improves cure rates, reduces disease transmission, and minimizes the risk of drug resistance, justifying its continued use in the country^62^ ^,^ ^63^. 

Beyond the traditional DOT model, Brazil has invested in personalized treatment approaches, recognizing that different population groups may benefit from alternative strategies. These include hybrid models that combine DOT with mobile technology for remote monitoring and self-administration support, thus offering greater flexibility for patients while maintaining treatment effectiveness. Recent analyses have further identified specific vulnerable subgroups for which expanding DOT delivers the greatest return on investment, including people experiencing homelessness (number necessary to treat (NNT): 3.00), people who use drugs (NNT: 3.72), and incarcerated individuals (NNT: 6.86). Focusing on tailored individual-level DOT interventions for these high-impact groups can maximize both clinical outcomes and cost-effectiveness^62^ ^,^ ^64^ ^-^ ^66^.

In Brazil, DOT has also been integrated into social policies such as the Bolsa Família Program, strengthening patient support and increasing treatment adherence. This combination of clinical and social support is essential for reducing the TB burden and advancing towards the goal of disease elimination by 2030^62^ ^,^ ^64^.

### The Use of Artificial Intelligence in TB Care

In recent years, the use of artificial intelligence (AI) in healthcare has expanded, thus contributing to the improvement of TB diagnosis and surveillance. A notable example is the automated interpretation of chest X-rays using computer-aided detection (CAD) systems, which can be integrated into both conventional and portable imaging devices^67^. A recent study conducted in Brazilian prisons demonstrated the effectiveness of this technology in TB screening among the incarcerated population, highlighting its potential to reach vulnerable groups^68^. The implementation of CAD in the SUS can provide greater autonomy to nurses and pharmacists providing TPT by facilitating the exclusion of active TB without relying exclusively on physicians or radiologists. 

In addition, the integration of AI with advanced statistical methods has led to the development of innovative tools for improving both epidemiological monitoring and disease management. These innovations include clinical scoring systems designed to predict patient outcomes according to clinical and epidemiological characteristics as well as predictive modeling approaches that guide more effective interventions for TB control^62^ ^,^ ^64^ ^,^ ^69^ ^,^ ^70^. 

### Brazilian Research Output in TB Science

**FIGURE 2:**
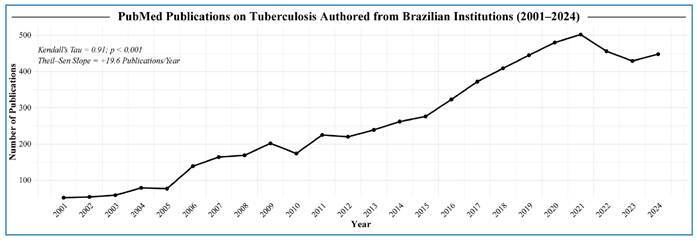
Annual PubMed-indexed tuberculosis publications from Brazilian institutions (2001-2024).

Legend: The line chart represents the total number of PubMed-indexed articles on tuberculosis-defined by the following search terms: “Tuberculosis” OR “Mycobacterium tuberculosis” OR (“TB” OR “Tuberculose” AND (Brasil [Affiliation] OR Brazil [Affiliation]), including at least one author affiliation in Brazil (per year from 2001 to 2024). The solid black line connects the annual counts, and the markers denote individual years. The inset text gives the Mann-Kendall trend statistic (τ = 0.91, p < 0.001) and the Theil-Sen slope (+19.6 publications/year), illustrating a strong, significant upward trend over the study period.

These innovations for TB control have been underpinned by parallel growth in research activity since REDE-TB was founded in 2001. TB research output from Brazilian institutions has surged by 761.5%, demonstrating a robust upward trajectory (Kendall’s tau = +0.91, p < 0.001) over the past two decades (Figure 2). Additionally, from 2002 to 2016, REDE-TB also facilitated the capacity building and training of over 2,000 healthcare professionals in TB research methods that can be applied in their workplaces^71^. National and international consortiums such as RePORT-Brazil have further accelerated both the volume and quality of TB science in Brazil, with collaborations between investigators resulting in 53 high-impact peer-reviewed publications since 2016^72^.

Nonetheless, after peaking at 503 publications in 2021, Brazil’s TB research output has steadily declined, falling to 480 in 2022 and 450 in 2023, with a modest rebound to 460 publications in 2024. This downturn mirrors wider shifts in research priorities during the COVID-19 era, and possibly reflects the 90% budget cut experienced by Brazilian scientists in 2021, with recuperation starting only in 2023. Furthermore, other emerging budgetary constraints and grant freezes have narrowed the window for early career investigators. Left unchecked, these converging trends threaten Brazil’s hard-won gains in TB science.

### Funding and Declining Interest in TB Research

Despite the impact of scientific advancements in TB control strategies, research in this field faces significant challenges related to the funding and training of a new generation of researchers. The decline in TB research investment reflects a global trend in which neglected diseases, including TB, receive insufficient funding compared to conditions with greater public visibility and commercial impact.

### Decline in TB Research Funding

Since the 1970s, following the introduction of a short-course TB treatment with rifampicin, isoniazid, and pyrazinamide, TB was widely perceived to be under control^3^ ^,^ ^73^. This misconception led to the closure of sanatoriums, dismantling of national TB control programs and nongovernmental organizations, and removal of TB education from the healthcare curriculum at universities. However, the resurgence of TB, particularly in association with the HIV/AIDS epidemic in the 1980s, prompted WHO to declare the disease a major public health issue in 1993^3^ ^,^ ^73^. Despite the launch of the Global Plan to Stop TB in 1996 and Global TB Elimination Plan in 2015, TB research continues to receive insufficient funding^74^.

Historically, scientific research investments have been associated with high economic and social returns in various countries. In the United States, every US dollar invested in basic research generates an estimated return of $4 to $9, driving technological innovation and healthcare economics^74^ ^,^ ^75^. Similarly, in the United Kingdom, the Royal Society estimates that every pound invested in science results in a return of £7 to £10, which stimulates growth in the biomedical sector^76^. In Brazil, a study found that investments in public health research yielded a return of R$1.61 in the Brazilian GDP, demonstrating the multiplicative positive economic impact of scientific research, with even further returns varying between R$7 and R$27.0 for research in other areas such as agriculture^77^ ^,^ ^78^.

Despite these promising returns, TB research remains underfunded. According to the Treatment Action Group (TAG), there is a global annual funding gap of over $1.3 billion for TB research and development (R&D), which represents less than half of the funds required to meet the WHO’s TB elimination goals by 2030^1^ ^,^ ^74^. While diseases such as cancer and cardiovascular conditions receive billions in funding^74^ ^,^ ^79^, TB remains neglected, despite being the leading cause of death among infectious diseases worldwide. 

Even with clear parallels in the global burden and mortality between COVID-19 and TB, the pandemic did not catalyze TB research funding. While COVID-19 has benefitted from an unprecedented, coordinated multinational and multisectoral response facilitating rapid diagnosis, vaccines, and treatment discoveries, TB investment has consistently fallen short of the annual target of $2 billion since 2018^74^. This gap reflects a troubling lack of urgency in addressing the longstanding TB pandemic, despite the recent resurgence of COVID-19 as the world’s biggest killer among infectious diseases^74^. 

Furthermore, recent severe cuts in TB care and research funding worldwide have drastically affected TB services in countries with high TB burden^80^. The suspension of research aid poses a severe risk of disrupting the recent gains made in highly affected countries worldwide^80^ ^,^ ^81^. Although Brazil’s national TB program budget currently comes solely from domestic funding, progressive cuts in public funding for scientific research in recent decades have severely undermined nationwide research efforts^1^ ^,^ ^82^. Since 2015, economic crises and political shifts have significantly impacted budgets for institutions such as the CNPq and the Brazilian Federal Agency for Support and Evaluation of Graduate Education (CAPES). In 2021, the Ministry of Science, Technology and Innovations (MCTI) faced a budget cut of R$ 600 million, which directly affected research grants and strategic projects^82^. However, in the last two years, efforts have been made to restore investments in science and technology, with new funding calls focused on neglected diseases as part of the CIEDDS and the *“Brasil Saudável”* (Healthy Brazil) action plan^13^. Despite these efforts, significant funding gaps remain, leaving many priority areas underfunded^83^. 

Furthermore, in 2023, the “National Strategy for the Development of the Health Economic and Industrial Complex” was officially launched by the Federal Government with an expected investment of R$42.1 billion by 2026. The strategy comprises six structuring programs and aims to expand the national production of priority items for the Unified Health System and reduce Brazil’s dependence on foreign supplies, medicines, vaccines, and other health products^84^. 

Nonetheless, recent funding cuts by major international agencies, including the National Institutes of Health and USAID, threaten the national and international scientific momentum. Currently, many Brazilian researchers who require extramural grants face critical funding shortages that jeopardize both ongoing national projects and collaborative international initiatives^85^ ^,^ ^86^. 

These subsequent funding reductions directly affect the training of new generations of scientists, particularly in research on neglected diseases, such as TB. The lack of incentives has driven many young researchers to pursue more lucrative careers in the private sector or in clinical practice, resulting in a vicious cycle of low innovation and reduced impact of TB control strategies in Brazil.

### Declining Interest Among New Generations of Scientific Researchers

**FIGURE 3:**
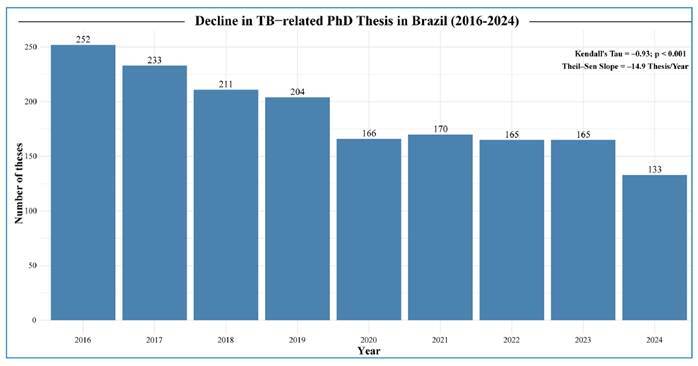
Decline in annual TB-focused PhD theses in Brazil (2016-2024).

Legend: Annual counts of completed PhD theses in Brazil containing the terms “Tuberculose OR Mycobacterium tuberculosis OR Tuberculosis” (2016-2024), with results from Theil-Sen and Mann-Kendall tests for trend calculations. The bars indicate the total number of theses per year (values are shown above each bar). Trend analysis presents a constant declining Theil-Sen slope estimate (-14.9 theses/year), illustrating a steady decline across the period. Mann-Kendall test confirms a strong, significant negative temporal trend (τ = -0.93, p = 7.95 × 10^−4^). Overall, the number of theses fell by 47.2%, from 252 in 2016 to 133 in 2024. Data were collected from the Brazilian Digital Library of Theses and Dissertations^19^.

The lack of financial and institutional incentives has discouraged the training of new generations of scientists in the field of TB. As shown in Figure 3, a constant and substantial decline has been observed in the number of completed TB-related PhD theses in Brazil since 2016. For many young scientists, the transition from completing a master’s or doctorate degree to securing independent funding is one of the greatest challenges in their academic careers. Limited funding coupled with the preference for grants that benefit established researchers at major institutions, make it difficult for new professionals, especially early-career researchers, to enter the field and reduces their interest in this area of study^87^. If unaddressed, this trend threatens to reduce Brazil’s contribution to the global scientific landscape as well as the country’s hard road towards a TB-free future. 

Additionally, the scarcity of formal research positions in the TB sector has exacerbated the crisis. While clinicians have ample opportunities in both the public and private sectors, public health specialists, epidemiologists, TB researchers, and similar professionals, struggle to find stable career paths. The salary disparity between these fields also influences career choices, making clinical practice financially more attractive than academic paths^87^.

Furthermore, the prioritization of emerging diseases with greater economic impact has diverted the attention of policymakers and researchers to other areas, leading to a decline in interest in TB research. The COVID-19 pandemic has highlighted this trend, as resources and projects have been redirected to SARS-CoV-2 studies, further marginalizing TB science. Without structured investments that create opportunities for young scientists, the continuity of TB research is at risk, potentially hindering long-term advancements in the fight against this disease.

Another critical challenge is the declining interest of young researchers in TB research. Although TB is a major global public health issue, it does not attract the same attention as oncological and cardiovascular diseases^74^, which offer more funding opportunities and professional development prospects. The lack of incentives, combined with decreasing research funding and bureaucratic hurdles in securing grants, has resulted in fewer researchers dedicated to studying TB.

Moreover, the inherently prolonged timelines of TB clinical research serve as a possible barrier to developing interest among young scientists. TB clinical research often spans several years, requires large cohorts with non-inferiority designs (given the high cure rates of existing drug-susceptible treatments), and commonly focuses on high-risk, hard-to-reach populations, demanding efforts that exceed standard PhD timelines and budgets, further discouraging early-career investigators^1^. To mitigate such issues, introducing early-career investigators within established platforms such as RePORT-Brazil can enable trainees to execute discrete, publishable subprojects within larger consortium studies.

### Proposals to Motivate a New Generation of Researchers

Combined with funding shortages, the declining interest in TB research calls for structured measures to attract and retain new scientists in the field. Reversing this trend requires a coordinated effort among governments, research institutions, universities, and the private sector to promote concrete incentives that ensure the continuity of scientific production and the renewal of TB research talent (Table 1).

**TABLE 1:** Key Challenges in Tuberculosis Research and Proposed Solutions.

Problems	Action Plans	Key Activities
Reduced public and private funding	Establish dedicated TB research funds	Launch Young Investigator TB Grants via CNPq/CAPES targeting regional TB challenges; co-fund with industry and NGOs; simplify FAPES calls for early-career applicants
Lack of interest among young researchers	Mentorship and curriculum integration	Recruit senior mentors from REDE-TB and academia; deliver workshops on grant-writing, study design, and publication; integrate TB modules and internships into undergraduate health programs
Lack of awareness and public engagement	Launch TB social-impact campaigns	Produce videos and infographics on TB’s burden; partner with scientists on social-media outreach and participate in accessible public science events
Lack of technological innovation	Accelerate TB R&D innovation	Establish public-private co-funding for new diagnostics and treatments; support startups and hackathons in key advancements including the use of AI and technology in TB care
Devaluation of scientific careers	Expand scholarships and awards for young TB scientists	Create early-career PhD/postdoc grants; offer travel awards to national and international TB conferences
Fragmentation of research networks	Strengthen national and international collaborations	Fund short lab exchanges between Brazilian and partner centers; form thematic working groups on high-priority TB-related topics; support collaborative pilot projects

Brazil has recently begun to recognize and reward young researchers differently in funding calls for neglected diseases, making competition fairer and promoting their long-term engagement in this field^87^. For instance, the Ministry of Health’s Department of Science and Technology (DECIT), together with CNPq and CAPES, has notably increased the TB research budget since 2021 (a 250% rise in state R&D funding)^83^. National calls, such as the “Socially Determined Diseases” CNPq/DECIT 2024 (R$40 million)^88^ and dedicated TB calls in 2023-2024 (R$20 million)^89^ offered opportunities for TB research funding in vast areas of the field. Additionally, state-level Foundations for Research and Innovation (FAPES) grants and postdoctoral scholarships for the fixation of Brazilian young doctors in 2023 were specifically aimed at critical public health problems from each state that could be solved through investments in R&D projects proposed by young Brazilian minds^89^. 

Nonetheless, this initiative still requires significant expansion, with more frequent calls for personnel, specifically for young investigators, offering them the ability to establish a research infrastructure and address critical public health questions. Scholarships and fellowships should be developed to further support early-career postdoctoral researchers, especially those aged <5 years after completing their PhDs (Table 1). 

Funding bodies, including the CNPq, CAPES, and state-level FAPES, could work to introduce “bridge grants” that finance pilot studies in neglected diseases such as TB, easing the transition from fellowship to independent funding. Concurrently, agencies could adapt models established by RePORT-Brazil’s REACT program as well as the UK NIHR Global Health Research Units model, which embeds training and mentorship opportunities into these grants through established researchers, creating a seamless pipeline from grant awards to capacity building^90^ ^,^ ^91^. In addition, grant-writing workshops and webinars could be offered to researchers at smaller institutions who often lack dedicated proposal support and the skills needed to compete successfully for funding opportunities. Moreover, policy changes that make research grants more accessible to young and independent researchers without requiring fixed affiliations with major institutions can help decentralize research and equalize access to funding.

By tailoring these awards to high-burden regions, each state’s FAPES grant calls can focus on locally relevant challenges addressed through new proposals to support the establishment of young doctors. For instance, in TB, several critical research priorities still need to be addressed, including developing and scaling new diagnostic and treatment technologies for remote areas, conducting genomic surveillance of drug-resistant strains to inform policy, innovating next-generation TB vaccines, and investigating social determinants that impede care for vulnerable populations. These are important research topics that can strengthen regional research capacity, while also accelerating solutions to pressing public health obstacles^15^ ^,^ ^92^. To address budgetary constraints, pilot programs could be co-funded with private partners or designed with scalable grant tiers to ensure sustainable support even in resource-limited states^91^.

Additionally, international exchange opportunities and participation in global research networks such as RePORT-Brazil and REDE-TB^91^ ^,^ ^93^ increase the visibility of early-career scientists and expand their opportunities for global collaboration. A common trajectory for young investigators also includes pursuing funding from the Wellcome Trust Early-Career Awards and the TWAS Research Grants^87^; by emulating these models and partnering with institutions implementing the models, Brazilian foundations can further extend career-development pathways for emerging TB scientists. Other notable examples, such as targeted grant streams (e.g., TrueNAT diagnostics) from India’s TB Research Consortium’s and European TB fellowships in sub-Saharan Africa, illustrate the impact of coordinated cross-border support^94^ ^,^ ^95^

Promulgating the view of scientific research as a tool for social impact is also crucial. TB is often perceived as a disease of the past or one that affects only marginalized populations, leading to reducing academic interest and public awareness. Integrating TB studies into undergraduate health curricula can increase students’ familiarity with the disease and encourage interest in research. At the same time, scientific outreach campaigns and incentives for researchers to engage with traditional digital media can help mobilize society and policymakers to prioritize TB research funding (Table 1).

Another critical focus area is securing public and private funding. The creation of dedicated TB research funds and the promotion of public-private partnerships are viable solutions for ensuring continuous financial support. These partnerships can sponsor infrastructure grants for university laboratories, thereby enabling the creation of state-of-the-art TB research facilities. Collaborations between academia, industry, and the government can overcome chronic underfunding for fast-tracking new TB technologies, which serves as a critical barrier towards developing novel drug regimens and diagnostic methods (Table 1).

A prime illustration is the Critical Path to Tuberculosis Drug Regimens initiative, which uses public-private collaborations to assist in developing and approving bedaquiline and pretomanid for TB use. To prevent common misalignments between academic and commercial timetables, codefined, flexible project milestones can be established with regular joint-committee reviews to synchronize the objectives.

Achieving sustained funding for TB research also requires strong political commitment. An example in Brazil is the Parliamentary Front to End Tuberculosis established in 2012 as a coalition of federal deputies dedicated to addressing TB as a public health priority. By facilitating discussions on strategic initiatives to strengthen the National Plan to eliminate TB by 2030, the Parliamentary Front also defended increased funding for research and innovation, particularly through parliamentary amendments, reinforcing the vital role of legislative support in the fight against TB^93^ ^,^ ^96^. 

Encouraging the use of emerging technologies is essential for making TB research more attractive and efficient. For example, AI-driven CAD, which demonstrates high accuracy in chest X-ray interpretation, requires rigorous field validation in remote and resource-limited settings to confirm its real-world performance^97^. Rigorous field trials using such technologies could promote TB screening in areas lacking specialist clinicians. Nanotechnology likewise offers breakthrough potential according to a recent FAPES-funded study which showed that antimicrobial peptides grafted onto antibiotic-loaded nanoparticles can re-sensitize multidrug-resistant *M. tuberculosis* in vitro, opening pathways for drug revitalization and targeted delivery^98^. 

Notably, integrating TB research with contemporary challenges, such as antimicrobial resistance (AMR) and the impact of climate change, has further broadened its appeal. Given that rifampicin-resistant and multiDR-TB account for a substantial share of global AMR mortality, joint funding calls that specifically reward projects at the TB-AMR interface can incentivize AMR specialists to pivot into TB science^99^. Similarly, fellowships that enable AMR researchers to rotate through TB laboratories would foster interdisciplinary collaboration. Aligning TB research with these high-priority themes and partnering with R&D technology firms that combine public and private funding can create compelling career pathways that attract young scientists and catalyze advancements in both TB control and other critical public health challenges (Table 1). 

Finally, international examples demonstrate the impact of scientific and social mobilization on prioritizing and valuing research. An important case study is the writer and activist John Green, who has used his platform to raise global awareness about TB and advocates for greater investment in research and treatment^100^. Similar models could be adapted in Brazil to strengthen science advocacy while working alongside civil society to foster greater political and institutional commitment to TB eradication.

Implementing such strategies is crucial to ensure that the progress made thus far is maintained and expanded. Strengthening TB research depends on talent renewal, increased funding, and recognition of science as an essential tool for promoting public health. Only through coordinated and sustained efforts, we can reverse the decline in research interest and accelerate progress toward TB elimination.

## CONCLUSION

Brazil has a rich tradition of TB research and control. However, it faces critical challenges in renewing its scientific workforce. Without swift, decisive action, Brazil risks hindering the advancements needed for a TB-free future. To ensure continued progress, we proposed a phased strategy across different time horizons. 

**Short-term recommendations**: Interest among early-career researchers should be stimulated by expanding targeted funding, strengthening mentorship networks, and integrating TB-related content into health science training. 

**Medium-term recommendations:** Institutional capacity should be consolidated through sustained investments, national and international collaborations fostered, and public-private partnerships promoted alongside initiatives to raise societal and political awareness.

**Long-term strategies:** Structural reforms that support the development of a new generation of senior TB scientists, including stable funding mechanisms, creation of career-track research positions, and alignment of TB research with broader health priorities, such as AMR and climate-related challenges, should be secured.

Therefore, we urgently call on government bodies, funding agencies, academic institutions, and research networks to adopt these strategies and empower emerging researchers to develop their careers in TB science.

## Data Availability

Data Availability Statement: Research data is not available.
